# Enhanced Natural Attenuation of Gasoline Contaminants in Groundwater: Applications and Challenges of Nitrate-Stimulating Substances

**DOI:** 10.3390/microorganisms13071575

**Published:** 2025-07-04

**Authors:** Zhuo Ning, Jiaqing Liang, Jinjin Ti, Min Zhang, Chao Cai

**Affiliations:** 1 Institute of Hydrogeology and Environmental Geology, Chinese Academy of Geological Sciences, Shijiazhuang 050061, China; ningzhuozhuo@163.com (Z.N.);; 2 Key Laboratory of Groundwater Remediation of Hebei Province & China Geological Survey, No. 92 Zhongshandong Road, Zhengding 050083, China; 3 State Key Laboratory for Ecological Security of Regions and Cities, Institute of Urban Environment, Chinese Academy of Sciences, Xiamen 361021, China; jqliang@iue.ac.cn

**Keywords:** enhanced natural attenuation (ENA), petroleum contamination, groundwater, nitrate, metagenome

## Abstract

Nitrate is a promising enhanced natural attenuation (ENA) material that enhances the microbial degradation of petroleum hydrocarbons by acting as an electron acceptor and nitrogen source. This study evaluated nitrate-containing materials (yeast extract, compound nitrogen fertilizer, and nitrate solutions) in microcosm experiments using gasoline-contaminated aquifer soils. Chemical analysis revealed that yeast extract achieved the highest degradation rate (34.33 mg/(kg·d)), reducing 600 mg/kg of petroleum hydrocarbons to undetectable levels within 18 days. Nitrate materials significantly increased nitrate-reducing activity and upregulated both aerobic/anaerobic hydrocarbon degradation genes, expanding microbial degradation potential. Metagenomic analysis identified *Pseudomonas* and *Achromobacter* as dominant genera across treatments, suggesting their critical roles in biodegradation. These findings demonstrate that nitrate-enhanced strategies effectively accelerate hydrocarbon attenuation under facultative anaerobic conditions, offering practical ENA solutions for petroleum-polluted sites.

## 1. Introduction

In industrialization and urbanization, petroleum and its products leak from various equipment, such as oil tanks and transportation pipelines, inflicting long-term and far-reaching damage on the groundwater ecological system [1,2,3]. Existing groundwater contamination treatment methods like multi-phase extraction, pump-and-treat methods, chemical oxidation, and a permeable reactive barrier (PRB) have issues including high costs, and a tendency to disrupt the ecological equilibrium of groundwater and cause secondary contamination [4,5]. Consequently, it is highly imperative to search for advanced technology that has a low treatment cost and is environmentally friendly.

Natural attenuation (NA) technology is grounded on the self-purifying capability of the aquifer, and accomplishes the harmless elimination of petroleum contaminants in the groundwater environment through natural processes such as biodegradation, convection, dispersion, dilution, adsorption, precipitation, and volatilization [6,7,8]. Among these, microorganisms use oxides as electron acceptors and convert petroleum hydrocarbons as electron donors into non-toxic or less-toxic substances such as CO_2_ [9,10,11]. However, the types and activities of microorganisms, as well as the electron acceptors, nitrogen sources, environment, and other conditions required for growth and metabolism, can limit the effect of NA, resulting in low efficiency and long periods of remediation [12,13,14]. Therefore, through the exploration carried out by many researchers, the concept of enhanced natural attenuation (ENA) has gradually been proposed [15]. Among these processes, biostimulation accelerates the biodegradation rate of pollutants through the addition of electron acceptors, nutrients, or biosurfactants [16,17]. In the field site, generally, hydrocarbons can be considered to be excessive in the plume, and microbial metabolism activity mainly depends on the supply of electron acceptors [18]. Thus, the addition of electron acceptors is considered to be a powerful means to promote microbial degradation activity.

Previous research has shown that the lower the energy required for an electron acceptor reaction is, the higher its reaction priority, which includes oxygen, nitrate, ferric iron (Fe (III)), and sulfate [19,20]. As the most preferred electron acceptor, oxygen is widely used in the remediation of petroleum in media such as soil through techniques such as bioventing [21]. However, in the issue of groundwater contamination, it is challenging for oxygen to enter the aquifer owing to the low solubility, and the use of aeration technology and slow-release materials also has the disadvantage of a limited scope of impact [22,23]. Fe (III) is insoluble with a low diffusion capacity in groundwater, and can lead to secondary contamination [24,25]. The final product of sulfate reduction is the sulfide ion, which also causes secondary contamination problems [26]. Nitrate as a stimulating material offers advantages such as low reaction energy, high solubility, not being readily adsorbed by soil particles, and being easily translocated to all parts of the aquifer [27]. The oxidation capacity of nitrate, which is second only to that of oxygen, can rapidly degrade contaminants, and the final product is non-toxic nitrogen gas (N_2_) [28]. Simultaneously, nitrate can also function as a high-quality nitrogen source while acting as an electron acceptor [29], thereby avoiding the secondary contamination of groundwater caused by the introduction of other nitrogen sources (such as ammonium) [30]. Furthermore, groundwater is predominantly a facultative anaerobic environment in most petroleum contamination plumes, wherein the flora mainly consists of facultative aerobic bacteria and nitrate-reducing microorganisms [31]. Therefore, the selection of nitrate as a stimulating material is in line with the redox conditions of most petroleum hydrocarbon-contaminated sites.

To screen nitrate-containing stimulants for petroleum hydrocarbon degradation, this study focused on aquifer soil–water media contaminated with gasoline, the most common petroleum product. Yeast extract powder, compound nitrogen fertilizer, and nitrate nutrient solution were selected as nitrate supply materials, NA and oxygen supply calcium peroxide were used as controls, and the optimal electron acceptor enhancing materials were selected through batch experiments. The promotion effect of nitrate-containing materials on the degradation activity of native microorganisms was analyzed through chemical and microbial metagenomic analysis. This work provided technical support for the remediation of oil-polluted groundwater.

## 2. Materials and Methods

### 2.1. Experimental Material

The aquifer soil used for carrying out the batch experiment was collected from a petroleum-contaminated site located in northwest China. The depth of the water table at the site ranges from 3.2 to 4.7 m below the ground surface. The lithology of the aquifer was mainly composed of fine sand. The aquifer soil was collected using a rotary drilling rig and stored in sterile resealable plastic bags at room temperature. The 92# gasoline was used as a petroleum contaminant in the study.

### 2.2. Experimental Design

Five different sets of experiments were conducted for 18 days in 40 mL brown bottles to study the efficiency of TPH degradation. The details of different treatment strategies are simulated in Table 1.

For each treatment strategy, three parallel samples were utilized to ensure sampling quality. In each set of experiments, 10 g of soil and different stimulators were added to the 40-milliliter brown bottles. To avoid damage to microorganisms from high salt concentrations, the ENA material was added in three separate doses. At the beginning, ENA materials with 2 milliliters of pure water were transferred into corresponding bottles, and the bottle caps were tightly screwed on. Then, 8 microliters of gasoline were injected into the bottle to provide the electron donor of the reaction. The brown bottle was incubated in the dark at 30 °C. Concentrations of gasoline, NO_3_^−^, NO_2_^−^, NH_4_^+^ as well as the nucleic acid in the bottles were measured at regular intervals of 6 days for testing. After each sampling, ENA materials with 0.2 mL of water were again injected into the corresponding remaining bottles.

### 2.3. Physical and Chemical Parameter Analyses

The concentrations of gasoline were determined using gas chromatography/mass spectrometry (GC/MS) (Agilent 7890a-5975c) equipped with a RTX-624 capillary column according to USEPA method 524.3 [34]. The concentrations of NO_3_^−^, NO_2_^−^, and NH_4_^+^ in the degradation process were measured by a spectrophotometer (Shimadzu UV2550) according to the methods from the Agricultural Chemical Analysis of Soil [35].

### 2.4. DNA Extraction and Sequencing

Parallel culture soil samples were mixed. About 0.56~2.55 g of soils (See Table S2) were used for DNA extraction according to a previous study [36]. After the DNA quality was checked, the DNA was fragmented, and a paired-end library was constructed as described in a previous study for shotgun metagenomic sequencing [37,38]. All sequencing was performed on an Illumina HiSeq4000 platform (Illumina Inc., San Diego, CA, USA) from Majorbio Bio-Pharm Technology Co., Ltd. (Shanghai, China).

### 2.5. Data Processing

(1) The concentration–time curves were plotted using Origin software (Pro 8 SRo).
(2) The apparent consumption concentrations of nitrate, nitrite, and ammonium were calculated using the added concentrations minus the detected concentration. A positive value signifies consumption and a negative value indicates generation, that is, the apparent generation quantity.
(3) The CH was taken as a petroleum hydrocarbon to calculate the stoichiometric relationship between the reduction amounts of oxygen and nitrate and the degradation amount of petroleum hydrocarbons [39,40]. The reduction amount of oxygen corresponding to the complete degradation of 1 mg/kg of CH substance is 3.08 mg/kg, and the reduction amount of nitrate is 4.77 mg/kg. It should be noted that the oxygen in the experiment bottle was calculated to be 8.57 mg, and the corresponding amount of degraded CH is 2.78 mg (that is, the corresponding concentration is 278 mg/kg).
(4) For microbial sequencing data, focus on the gene abundance data related to nitrogen transformation and hydrocarbon degradation. In the KEGG database, screen nitrate reductase genes, hydrocarbon oxygenase genes, genes related to benzene degradation, as well as other functional genes related to the metabolism of carbon, nitrogen, phosphorus, and sulfur. Gene abundance is expressed in ppm, that is, the number of reads containing this gene per one million reads [41]. Calculate the ratio of the abundance value of each gene sampled at different time points to the abundance value of the gene at time zero [32], and draw heat maps to evaluate the change in the relevant genes over time. The overall microbial population structure, as well as the structure of microbial populations containing certain key enzymes, was identified through comparison with the NR database and presented using bar charts [42].

## 3. Results and Discussion

### 3.1. Contaminant Degradation Characteristics

**Figure 1:**
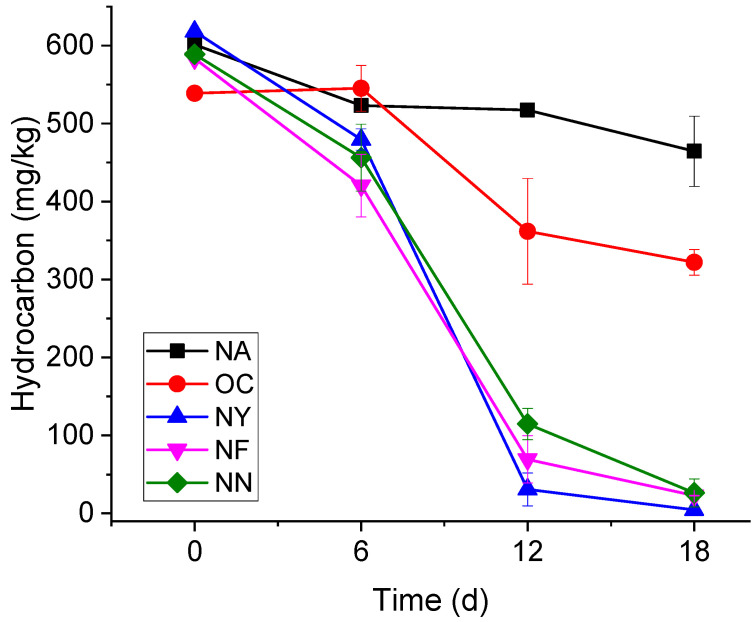
The variation in the gasoline concentrations over time.

The variation curves of the gasoline concentrations over time are shown in Figure 1. The NA group did not receive any chemical additives, and the concentration of contaminants showed a gradual decrease over time, but the process was relatively slow. Specifically, the initial concentrations of gasoline were approximately 600 mg/kg, and after 6 days, 12 days, and 18 days, the concentrations decreased to 523 ± 7 mg/kg, 517 ± 5 mg/kg, and 464 ± 45 mg/kg, respectively. After 18 days of self-degradation, on average, 23.4% of gasoline can be eliminated, and the degradation rate was around 7.78 mg/(kg·d). The results show that the aquifer had a certain degree of self-repair but at slow velocities. The initial gasoline concentrations of the OC group were about 540 mg/kg, and dropped to 322 ± 16 mg/kg in 18 days. The removal rate was 40.7%, and the degradation rate was only slightly higher than that of the control group. When CaO_2_ encounters water in the aquifer, it releases oxygen and OH^−^ ions [43]. Even though the pH of the OC group was controlled to not exceed 8.5 during the experiment, the pH of the OC group was still higher than that of the other groups. Although it provided the electron acceptor dissolved oxygen (DO), the altered environment made it difficult to maintain the original microbial metabolism [44].

Yeast extract powder (NY), nitrogen fertilizer (NF), and a nitrogen–phosphorus mixture (NN) can all provide microorganisms with various nutrients and nitrates. On the 18th day, gasoline concentrations in all three groups fell below 25 mg/kg, showing no significant differences (*p* < 0.05) among them, but significant differences (*p* < 0.05) emerged when compared with the other two groups (OC and NA). On average, the degradation rate of the NY group was as high as 34.33 mg/(kg·d), and the concentration of gasoline contaminants dropped to the detection limit. The gasoline concentrations in the NF group dropped from 580 mg/kg to 23 mg/kg, and the degradation rate was about 31.17 mg/(kg·d). The degradation rate of the NN group was slightly slower at 30.83 mg/(kg·d). The differences among these three can be attributed to the fact that yeast extract is derived from yeast cells. In addition to providing nitrate as an electron acceptor and essential macronutrients, yeast extract serves as a rich source of growth factors (e.g., B vitamins like niacin for NAD+ synthesis, and essential amino acids such as cysteine for metalloenzyme activation). The various amino acids, vitamins, and other nutrients it contains are closer to the actual requirements of microorganisms [45]. B vitamins (e.g., thiamine, niacin) act as coenzymes for electron transport chains in denitrifying bacteria; essential amino acids (e.g., tryptophan, lysine) are utilized for the rapid biomass synthesis of dominant genera like *Pseudomonas* and *Achromobacter*; nucleotides and trace elements activate key enzymes in both aerobic and anaerobic hydrocarbon degradation pathways. This synergistic provision of growth factors explains the muti-fold upregulation of anaerobic degradation genes (e.g., EC 4.1.99.11) observed in the NY group (Section 3.3.1), which is not fully replicated by synthetic nutrient formulations.

Overall, the addition of nitrates, particularly yeast extract, enhanced the capacity and rate of gasoline degradation, making them the preferred materials for the remediation of site aquifers.

### 3.2. Characteristics of Concentration Changes in Inorganic Nitrogen

**Figure 2:**
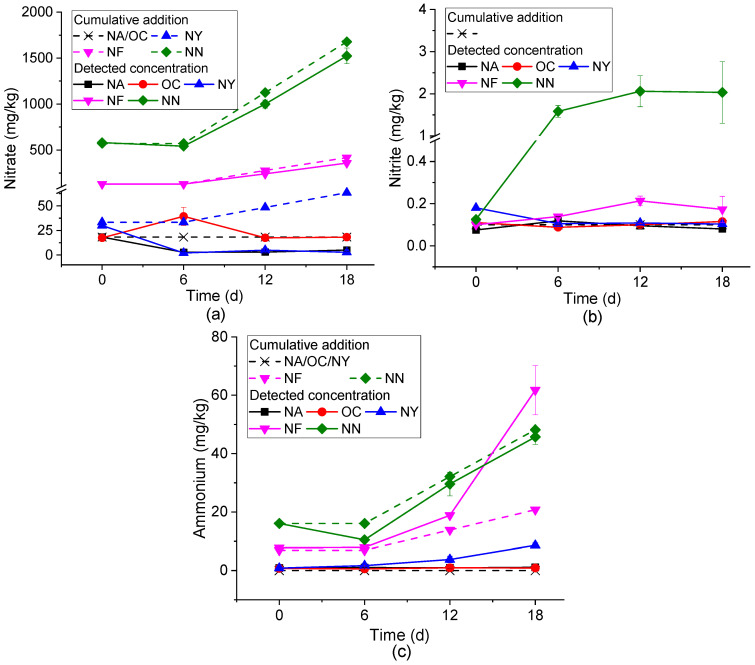
Inorganic nitrogen concentration variations in different ENA materials. Plots (**a**–**c**) represent nitrate, nitrite, and ammonium, respectively.

As shown in Figure 2, the mechanisms of action of each ENA material were analyzed through the changes in the concentrations of NO_3_^−^, NO_2_^−^, and NH_4_^+^ over time.

Figure 2a shows the concentration changes of NO_3_^−^. At the initial stage of the experiment (0 days), due to different stimulating materials, there was a large difference in the initial NO_3_^−^ content. By the sixth day of the reaction, except for a slight increase in the OC group, the concentrations of the other treatment groups all decreased, and the NA group dropped below the detection limit. In the subsequent reaction, except for the NA group, the consumption of NO_3_^−^ in the other groups gradually increased. In the subsequent period (from 6 to 18d), except for the NA group, the differences between the nitrate concentrations and the amounts added (corresponding to nitrate consumption) gradually increased in the other groups. The consumption of nitrate was generally due to nitrate reduction, where nitrate serves as an electron acceptor and reacts with an electron donor (such as petroleum hydrocarbons) under microbial action to undergo redox reactions, reducing to nitrite, nitrous oxide, nitrogen, or ammonium. During this process, electron donors like petroleum hydrocarbons release electrons and undergo oxidative degradation [46]. In the OC group, the NO_3_^−^ concentration increased on the sixth day. This is because the slow-release oxygen of calcium peroxide triggered nitrification in the system and converted nitrogen in the system into NO_3_^−^. Subsequently, in an aerobic environment, microorganisms use oxygen and NO_3_^−^ as the main electron acceptors to degrade petroleum hydrocarbons, resulting in a decrease in the concentrations of NO_3_^−^ and petroleum hydrocarbons [47]. The concentration of contaminants in the NA group only decreased significantly from 0 to 6 days, and there was no obvious change in the subsequent concentration, which was the same as the change in NO_3_^−^ concentration. This confirmed that the nitrate reduction process was involved in the NA process.

Figure 2b shows the concentration change of NO_2_^−^. The NN group increased from about 0.1 mg/kg on day 0 to 2 mg/kg on days 6–18, indicating that secondary pollution caused by NO_2_^−^ accumulation should be paid attention to when NN is applied. The concentration fluctuations in other groups were small. Among them, from the perspective of the average value, the NY group showed an overall downward trend, the NF group showed an upward trend, the NA group first increased and then decreased, and the OC group first decreased and then increased. However, there were no statistically significant differences (*p* < 0.05) in the changes observed for NY, OC, and NA groups between 6 and 18 days. The intermediate product NO_2_^−^ may be produced in both the processes of nitrate reduction and NH_4_^+^ oxidation. The systems other than OC were anaerobic environments. Therefore, the NO_2_^−^ in the NN, NF, and NA systems is speculated to come from the nitrate reduction process, indirectly indicating the occurrence of the nitrate reduction and degradation process. The NO_2_^−^ in OC may be derived from the ammonization of soil nitrogen and the subsequent nitrification process in the system. The yeast extract in the NY group is the preferred energy source, carbon source, and nitrogen source for most microorganisms [48], making it difficult for the intermediate product NO_2_^−^ to accumulate. The accumulation of NO_2_^−^ generated during the ENA process can lead to the secondary pollution of groundwater. Therefore, materials that can cause nitrite accumulation, particularly those in the NN group, need to be further optimized.

Figure 2c shows the concentration change of NH_4_^+^. There was basically no change in NA and OC (*p* < 0.05). The difference between the measured value and the total added amount of the NN group reaches the maximum at 6 days, and then decreases and remains stable. This may be caused by the consumption of NH_4_^+^ as a nitrogen source for microbial growth, and then ammonia was produced due to ammonification. NY and NF show an overall upward trend, and the growth was obvious after 6 days. These two groups of systems contain organic nitrogen, and ammonification might occur, resulting in an increase in NH_4_^+^ concentration [49]. The production of high-ammonia nitrogen will lead to the eutrophication of water, indicating that the dosage of NF and NN needs to be strictly controlled, especially as NF will produce a large amount of ammonia nitrogen in the later stage of use.

Based on the typical reaction of nitrate reduction for the degradation of petroleum hydrocarbons [50], the hydrocarbon degradation amount corresponding to nitrate consumption can be estimated. The results are shown in Table 2. Comparing the actual degradation amount (AD) with the theoretical degradation amount (TD), it is found that the TD value was less than one-tenth of the AD value. The reasons include mainly two aspects: First, oxygen in the culture system preferentially participates in the hydrocarbon degradation reaction as an electron acceptor [51]. However, the amount of gasoline degradation corresponding to oxygen in the sealed culture flask is only 278 mg/kg. After deducting this value, the actual degradation amount of the NY, NF, and NN groups is still too large. Second, petroleum hydrocarbons undergo incomplete degradation and are not completely mineralized into CO_2_, but exist in the form of intermediates or intermediate states, resulting in deviations in the stoichiometric relationship. Thus, in the degradation effect evaluation, only evaluating the contaminant degradation amount through the consumption of electron acceptors may lead to underestimation [39,52]. On the other hand, paying attention to the reduction in contaminant concentration does not mean that the reduced part was completely degraded and may be converted into other intermediate products. Therefore, the appearance of intermediate products with enhanced toxicity needs to be prevented.

### 3.3. Microbial Functional Characteristics

#### 3.3.1. Hydrocarbon Degradation Functional Genes

**Figure 3:**
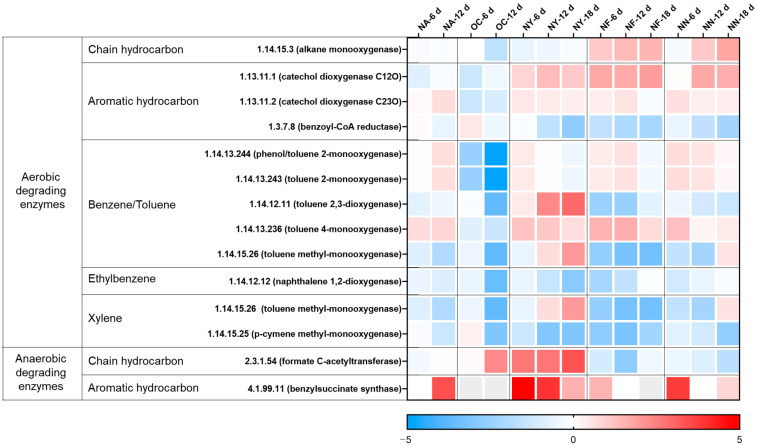
Heat map illustrating variations in the abundance of genes related to hydrocarbon degradation. The colors depicted in the figure correspond to the log_2_ ratios of gene abundance in the sample compared to the abundance initially (at the 0 time point).

The changes in the abundance of hydrocarbon degradation-related genes at various time points for different treatments are shown in Figure 3. The overall genes of the NA group were not much different from those before the reaction. However, the abundance of anaerobic degradation genes for aromatic hydrocarbons (4.1.99.11, benzylsuccinate synthase, BssA) markedly increased on the sixth day. On the 12th day, the abundance of some genes for the aerobic degradation of benzene series compounds rose, while the abundance of ethylbenzene and xylene oxidation degradation genes decreased. The soil samples for the experiment were collected and preserved in an anaerobic environment, but in this experiment, they were placed in a facultative environment with headspace air, and gasoline and sterile water were added. In the redox environment, moisture and contaminant concentrations change, leading to changes in the abundance of functional genes and activating NO_3_^−^ reduction anaerobic degradation functional genes. Thus, NO_3_^−^ was reduced and contaminants were degraded. When there was no NO_3_^−^, the contaminant concentrations ceased to decrease.

In the OC group, the abundance of each gene was significantly reduced, except for the gene encoding the chain hydrocarbon anaerobic degradation enzyme (EC 2.3.1.54, formate C-acetyltransferase). This is attributed to the fact that the incorporation of CaO_2_ served as an oxygen-releasing agent, which disturbed the original acid–base equilibrium, gave rise to an alkaline environment [43], and subsequently impacted the original ecological balance [43,53]. The microorganisms containing the gene (EC 2.3.1.54) in this system may be able to tolerate a certain level of alkalinity.

In the NY group, except for some aerobic degradation genes, the abundance of other genes increased notably, especially the two genes for anaerobic degradation, with the highest abundance increasing by 10 to 30 times. Yeast extract contains a large quantity of substances required by microorganisms and meets the growth needs of most microorganisms [48]. In the culture system under stress from petroleum contaminants, the relative abundances of microorganisms that adapt to petroleum contaminants and degrade petroleum hydrocarbons were raised.

The gene change tendencies of the NF group and the NN group were relatively congruent. In the aerobic degradation process, the increases in gene abundance were mainly focused on chain hydrocarbon degradation monooxygenase, aromatic hydrocarbon degradation catechol dioxygenase, and benzene/toluene degradation monooxygenase. In the anaerobic degradation process, the abundances of key genes for aromatic hydrocarbon degradation were raised. However, it cannot be inferred from this that aromatic hydrocarbons can be degraded through aerobic and anaerobic processes, while chain hydrocarbons are mostly degraded through aerobic action. Since the database of functional enzymes for petroleum hydrocarbon anaerobic degradation is incomplete [54], the key functional genes in anaerobic environments require further exploration.

In conclusion, all three nitrate materials can be operative in facultative environments. Specifically, nitrate can act as an electron acceptor to accelerate the anaerobic degradation of pollutants in the nitrate reduction process. Additionally, it can function as a nitrogen source to boost the growth of aerobic or anaerobic degradation microorganisms and promote both aerobic and anaerobic degradation. Concurrently, in contrast to other stimulating substances, yeast extract can conspicuously augment the abundance of key functional genes for petroleum hydrocarbon degradation, which can be reciprocally attested by the results of the degree of petroleum hydrocarbon degradation.

#### 3.3.2. Functional Genes Related to Nitrate Reduction

**Figure 4:**
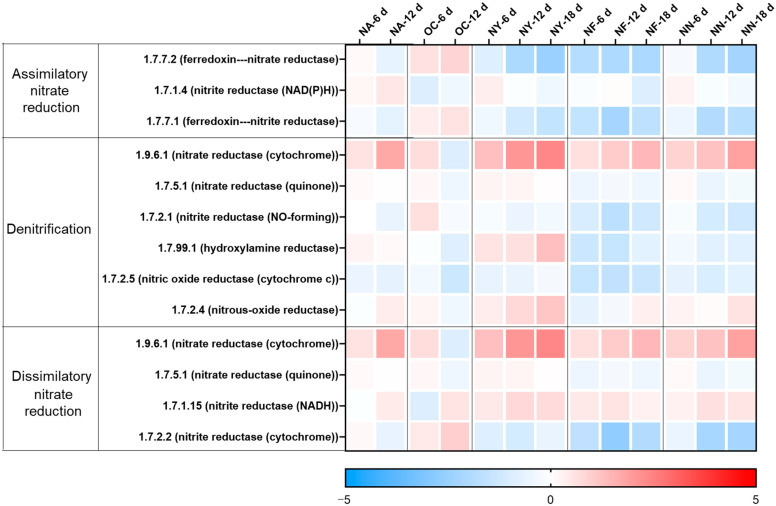
Heat map illustrating variations in the abundances of genes related to nitrate reduction. The colors depicted in the figure correspond to the log2 ratios of gene abundance in the sample compared to the abundance initially (at the 0 time point).

The changes in the abundance of nitrate reduction-related genes at various time points for different treatments are shown in Figure 4. As shown in the figure, in the NA group, only the gene for nitrate reductase (cytochrome) (EC 1.9.6.1), which is required for the first step of both denitrification and dissimilatory nitrate reduction, increased at 12 days, while the overall differences in the remaining genes were not significant compared to those before treatment. In the initial soil samples from this aquifer, there was a relatively large number of nitrate-reducing bacteria and a certain amount of nitrate, but a lack of petroleum contaminants as electron donors. The introduction of contaminants stimulated the growth of nitrate-reducing bacteria. However, the functional genes for the subsequent reduction of nitrite to nitrous oxide and nitrogen did not increase, but the detected concentration of nitrite was very low. This may be because the samples from the aquifer contained a sufficient amount of nitrite-reducing bacteria to reduce the limited nitrite, with the combined abundance of their functional genes encoding the enzymes of EC 1.7.2.1 and EC 1.7.99.1 exceeding 100 ppm, which was much greater than the gene abundance of 1.9.6.1 at 6 ppm.

In the OC group, the gene abundance slightly increased on the sixth day, but the overall change was not significant. On the 12th day, the abundance of nitrification functional genes decreased, and the abundance of genes for assimilatory and dissimilatory reduction to ammonium increased, which partly explained the increase in ammonium concentration.

The abundance of functional genes in the NY group witnessed a considerable increase, and was predominantly concentrated in the key enzyme genes of the denitrification process, facilitating microorganisms to reduce nitrate to nitrite and eventually to N_2_O or N_2_. Concurrently, the periplasmic nitrate reductase (Nap) is insensitive to oxygen molecules, enabling denitrification to occur under aerobic conditions. Aerobic denitrifying bacteria are typically heterotrophic bacteria, which can employ organic carbon as the carbon source under aerobic conditions and convert reduced nitrogen (inorganic nitrogen or organic nitrogen) into oxidized nitrogen. All these manifestations can suggest that yeast extract holds the potential to enhance the denitrification degradation of petroleum pollutants in the petroleum-contaminated aquifer, which is in accordance with the analysis results of TPH degradation efficiency and the key functional genes of petroleum hydrocarbon degradation. The results of the NF and NN groups are relatively consistent. The abundance of some key enzyme genes, including the conversion of nitrate to nitrite (EC 1.9.6.1), the nitrous oxide to nitrogen (EC 1.7.2.4), and the dissimilatory reduction of nitrite to ammonium (EC 1.7.1.15), for nitrate reduction, increased. However, the degree was not as high as that of the NY group, and the stimulation of the key enzyme for the conversion of nitrite to nitric oxide was missing, resulting in nitrite accumulation.

Therefore, the analysis results of nitrate reduction-related functional genes and the analysis results of key functional genes for hydrocarbon degradation mutually corroborate each other, confirming that yeast extract can indeed stimulate the metabolism of nitrate-reducing hydrocarbon-degrading microorganisms and has the potential to enhance their petroleum hydrocarbon degradation capacity. Both compound nitrogen fertilizer and self-prepared nitrogen-containing nutrient solution can also stimulate some nitrate-reducing microorganisms to exert their petroleum hydrocarbon degradation ability, but the range of applicable microorganisms may be narrower compared to yeast extract. Although calcium peroxide increases the abundance of some nitrate-reducing environmental microorganisms, it reduces the total number of microorganisms (as indicated by the lower amount of extracted DNA; see Table S2) and fails to significantly increase the abundance of key hydrocarbon degradation genes, resulting in limited enhancement effects. Furthermore, the nitrate reduction genes of NY were well enriched, yet the NF and NN groups did not display enrichment levels surpassing those of NA and OC. This is on account of the fact that the experimental soil originated from a nitrate environment and could multiply in an appropriate culturing environment. Nevertheless, different groups stimulated diverse types of microorganisms (see the microbial analysis), thereby resulting in distinct capabilities for nitrate reduction.

Overall, the analysis results of hydrocarbon degradation genes and nitrate reduction genes are consistent with the degree of petroleum hydrocarbon degradation, all indicating that nitrate-containing materials are effective ENA materials, and that yeast extract may be the optimal choice.

#### 3.3.3. Other Enhanced Functional Genes

The abundance of some functional genes related to nitrogen, phosphorus, and sulfur metabolism also changed after the application of ENA (Table S3). In different groups, specific genes were enriched (Table S3). These specific genes play different roles in petroleum hydrocarbon degradation. In the NF group, enriched formaldehyde dehydrogenase (EC 1.2.1.46) contributes to oxidizing degradation-generated aldehydes, and the ABC-type taurine transporter (EC 7.6.2.7) influences sulfur utilization by transporting sulfur-containing compounds [55]. In the NY group, alpha-D-ribose 1-methylphosphonate 5-phosphate C-P-lyase (EC 4.7.1.1) accelerates the decomposition of phosphorus-containing portion in petroleum hydrocarbons [56]. In the NN group, 3-phytase (EC 3.1.3.8) provides phosphorus nutrition by hydrolyzing organic phosphorus, and ribose 1,5-bisphosphate phosphokinase (EC 2.7.4.23) regulates phosphorus metabolism that is crucial for degradation energy and substance transformation [57].

In general, the stimulus genes identified in phosphorus and sulfur metabolism are mainly genes involved in the mineralization of organic elements. In particular, organophosphate mineralase genes such as 3-phytase (EC 3.1.3.8), ribose 1,5-bisphosphate phosphokinase (EC 2.7.4.23), alpha-D-ribose 1-methylphosphonate 5-triphosphate diphosphatase (EC 3.6.1.63), alpha-D-ribose 1-methylphosphonate 5-phosphate C-P-lyase (EC 4.7.1.1), and (aminoalkyl)phosphonate N-acetyltransferase (EC 2.3.1.280) are all involved in phosphonate utilization [58,59,60,61]. Phosphonates are a resource for microorganisms that live in environments with limited phosphate availability [62]. To take advantage of these resources in nutrient-scarce environments, bacteria have evolved systems to uptake and catabolize phosphorus compounds for their subsequent use as carbon and phosphate sources [61]. In addition, formaldehyde dehydrogenase (EC 1.2.1.46) could promote the dehydrogenation of aldehydes in the degradation of alkanes to form corresponding fatty acids that provide energy to cells [63]. The ABC-type taurine transporter (EC 7.6.2.7) is expressed under sulfate-starved conditions and can help bacteria use aliphatic sulfonates as a source of sulfur for growth [64].

Thus, these ENA materials can enhance the viability of microorganisms under oligotrophic conditions, indicating that these functions help microorganisms degrade contaminants. Moreover, the prominent enrichment of genes related to phosphonate utilization suggested that this may be an important stimulus point to promote the function of microorganisms in the presence of petroleum contamination.

### 3.4. Microbial Community Structure

#### 3.4.1. The Structural Characteristics of All Microbial Communities

**Figure 5:**
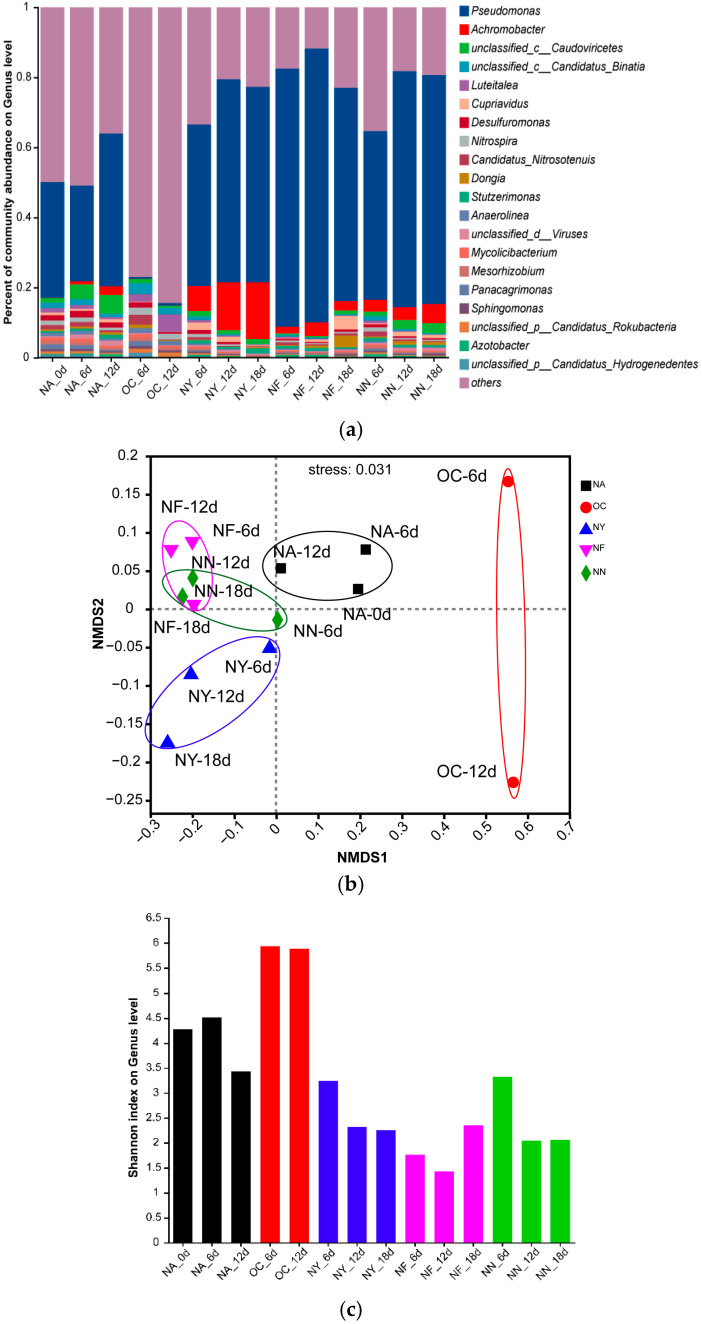
Bar plot (**a**), non-metric multidimensional scaling (NMDS) plot (**b**), and Shannon index plot (**c**) of the microbial community structure at the genus level. “Others” indicates species with an overall abundance of less than 0.01.

Figure 5 displays the changes in microbial community structure during the biostimulation process. From the NMDS plot (Figure 5b), it was observed that all samples from the ENA treatment groups deviated significantly from the NA group. Within each group, the sample points were relatively clustered, and the degree of deviation increased over time, with the most pronounced deviation occurring within the first 6 days. Different groups exhibited distinct deviation patterns: the OC group showed the largest deviation, shifting towards the positive direction on the NMDS1 axis, while the other groups demonstrated smaller deviations, shifting towards the negative direction on the NMDS1 axis. Additionally, the NY group gradually shifted towards the negative direction of the NMDS2 axis over time. This indicates that, overall, the ENA materials altered the microbial community structure, and the microbial community structure of the OC group differs significantly from that of the other groups, with this change occurring within the initial 6 days of addition. The specific differences can be seen in the bar plot (Figure 5a). *Pseudomonas* was the dominant genus in the original soil samples, with increased abundance in the NA, NY, NF, and NN groups, but it nearly disappeared in the OC group. Besides *Pseudomonas*, the abundance of *Achromobacter* increased after ENA in all groups except for OC, with the NY group showing the most significant increase. Both *Pseudomonas* and *Achromobacter* are common petroleum hydrocarbon-degrading bacteria [65,66]. These results suggest that the petroleum-contaminated aquifer originally contained a large number of microorganisms with potential for petroleum hydrocarbon degradation, and the abundance of these degrading microorganisms further increased under the stimulation of nitrate-containing materials which released their degradation potential. Additionally, “others” accounted for the majority in the OC group, indicating a surge in the proportion of microorganisms with low abundance. Shannon index (Figure 5c) analysis revealed that the microbial richness in the OC group increased, whereas the genus richness decreased in all other treatment groups. In addition, different treatments exhibited a distinct relative dominance of specific bacterial genera. For instance, in the OC group, the relative abundance of *Luteitalea* increased, although there have been no reports to date on its ability to degrade petroleum hydrocarbons. In the NF and NY treatments, *Cupriavidus* was relatively abundant, and this genus may possess the capability to degrade petroleum hydrocarbons [67]. The above evidence confirms that nitrate treatment can directionally domesticate microorganisms and promote pollutant degradation.

#### 3.4.2. Nitrate-Reducing Functional Microorganisms

**Figure 6:**
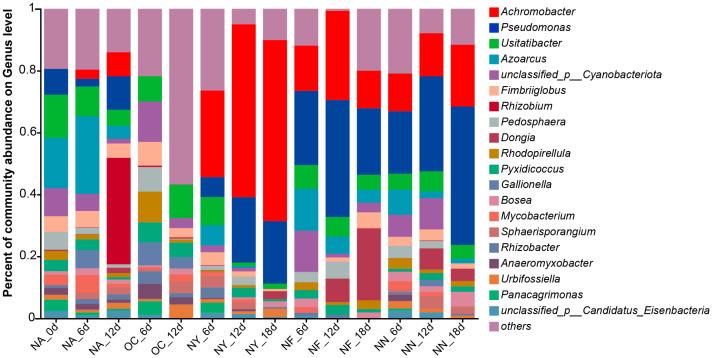
Genus-level abundance plot of microorganisms containing the encoding gene for nitrate reductase (cytochrome) (EC 1.9.6.1).

Figure 6 illustrates the abundance of microorganisms that possess the encoding gene for nitrate reductase (cytochrome) (EC 1.9.6.1), which is not only the very first step but also the most vital enzyme in the process of nitrate reduction. The results indicate that the abundance of *Pseudomonas* and *Achromobacter* increased significantly in the NY, NF, and NN treatment groups, with a slight increase in *Achromobacter* abundance in the NA group. This trend is consistent with the overall changes in microbial abundance. Some *Pseudomonas* species have been reported to perform a denitrifying degradation of petroleum hydrocarbons under anaerobic or aerobic conditions [68,69], while *Achromobacter* can perform a nitrate reduction under aerobic conditions [70], but this generally exists in anaerobic environments in petroleum hydrocarbon-contaminated environments [47,71]. Although the denitrifying petroleum hydrocarbon degradation functions in the species of these two genera discovered in this study (Figure S1) have not been previously reported, preliminary identification based on metagenomic information suggests that these microorganisms possess the potential for these functions, warranting further investigation for confirmation. This explains the increase in *Achromobacter* abundance in the NA group later on, possibly due to oxygen depletion in the system. The process of using nitrate as an electron acceptor and reducer is denitrification, which is the highest-energy-yielding respiration system in anoxic environments, and the number of microorganisms with a denitrification ability are known as denitrifying bacteria [72]. The accumulation of intermediates, such as NO_2_^−^, is prone to occur in the denitrification process and is unfavorable to the growth and metabolism of the bacteria in the denitrification process, which becomes a limiting step in the denitrification process [73]. This suggested that although NN also exhibited an excellent removal of petroleum hydrocarbon, the resulting increase in NO_2_^−^ concentration may limit its application.

In addition, a high amount of nitrate inhibits the growth of sulfate-reducing bacteria (SRB). SRB produce sulfide as an end-product of sulfate respiration, which is toxic to most organisms [74]. In oil wells, heterotrophic nitrate-reducing bacteria can compete with SRB for volatile fatty acid electron donors, further reducing the production of sulfide [75]. Therefore, nitrate stimulants can be used to control the growth of SRB and prevent sulfide production while promoting the degradation of petroleum hydrocarbons.

#### 3.4.3. Enriched Hydrocarbon-Degrading Microorganisms

**Figure 7:**
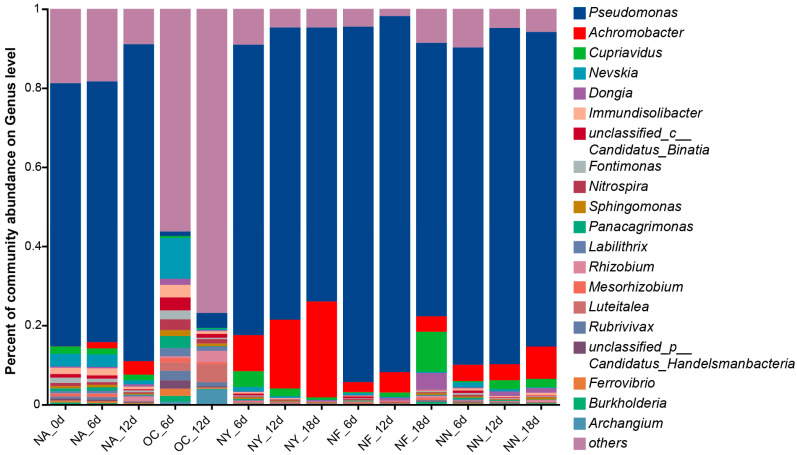
Genus-level abundance plot of microorganisms containing the encoding gene for the degradation key enzyme catechol 1,2-dioxygenase (EC 1.13.11.1).

Upon the addition of stimulants, the abundances of gene-encoding catechol 1,2-dioxygenase (EC 1.13.11.1) were significantly increased in all NY, NF, and NN groups. Microorganisms identified as possessing the representative aerobic degradation key enzyme EC 1.13.11.1 are shown in Figure 7. The results indicate that, except for the OC group, the dominant species containing the aerobic degradation EC 1.13.11.1 gene are still *Pseudomonas* and *Achromobacter*, with *Pseudomonas* occupying an absolute advantage (Figure 7). According to reports in the literature, these species can not only utilize nitrate as an electron acceptor but also utilize oxygen as an electron acceptor for hydrocarbon degradation [66,76]. These results suggested that ENA materials can still enhance the growth and degradation capacity of denitrifying bacteria under oxygen-containing conditions by enhancing nitrate respiration or providing nutrients such as nitrogen sources, despite the anoxic origin of these bacteria.

Microorganisms identified as possessing the representative anaerobic degradation key enzymes benzylsuccinate synthase (EC 4.1.99.11) and formate C-acetyltransferase (EC 2.3.1.54) are *Aromatoleum* and *Azoarcus*, and *Oscillochloris*, *Actinotalea*, and *Herbinix*, respectively (see Figure S2 for details), none of which are dominant species in the system. The bacterial members of the *Aromatoleum*/*Azoarcus* cluster are facultative anaerobic degradation specialists and a group of model organisms that study anaerobic BTEX degradation [77]. In particular, the genus *Aromatoleum* can degrade a wide range of aromatic compounds under anoxic denitrification conditions [78]. Phylogenetic clusters are widely distributed in terrestrial and aquatic habitats, including agricultural soils, contaminated sites, and wastewater treatment plants. *Actinotalea* has also been reported as a facultative anaerobic organotrophic bacterium and has been isolated from the composition of the anaerobic oil-degrading methanogenic enrichment obtained from an oil reservoir [79]. These bacteria indicated a higher potential for the anaerobic degradation of petroleum hydrocarbons in situ an oil-contaminated environment. *Oscillochloris* a mesophilic, filamentous, photoautotrophic, non-sulfuric, diazotrophic bacterium which is capable of carbon dioxide fixation via the reductive pentose phosphate cycle [80]. *Herbinix* is a Gram-positive anaerobic bacterium that exhibits a wide range of hemicellulose degradation capacities in thermophilic biogas reactors [81]. The presence of key anaerobic degradation genes indicated that these bacteria also have the potential to anaerobically degrade petroleum hydrocarbons, which deserve further attention.

Therefore, in this study, nitrate-containing stimulatory materials stimulated the growth of indigenous petroleum hydrocarbon-degrading dominant bacteria such as *Pseudomonas* and *Achromobacter*, which are facultative anaerobic microorganisms. These microorganisms can degrade petroleum hydrocarbons using oxygen or nitrate as electron acceptors under facultative oxygen conditions.

### 3.5. Implications for ENA Application

This study simulated the enhanced natural attenuation (ENA) process in a facultatively anaerobic environment, where widely distributed contamination plumes were exposed to several types of ENA materials. The results indicated that the addition of electron acceptors such as nitrate (NO_3_^−^) and nutrients was particularly crucial. In this study, there was little difference in the final contaminant degradation effects between artificially formulated nutrients (including complex nitrogen fertilizers and self-formulated nutrients) and natural yeast extract. However, during some ENA processes, there may be the generation of secondary pollutants such as more toxic or mobile intermediate degradation products of gasoline, as well as NO_2_^−^. We need to remain highly vigilant regarding this. In response, we should further identify the intermediate degradation products of gasoline during the ENA process, and evaluate their toxicity and mobility. Moreover, we should further optimize the material formulations to reduce the production of highly toxic pollutants. Additionally, we should monitor potential secondary pollutants throughout the ENA process to prevent the occurrence of secondary pollution.

In addition, microbial analysis revealed that the addition of yeast extract could stimulate more hydrocarbon-degrading functions, fully mobilizing microbial potential, which is particularly important for certain difficult-to-degrade microorganisms. However, when using yeast extract in actual field ENA applications, special attention should be paid to the following points: Firstly, yeast extract mostly exists as organic molecules [48], which may cause secondary groundwater contamination. Secondly, yeast extract is a complex mixture, and the migration and transformation processes of its components in actual groundwater are inconsistent [82], leading to the absence of certain key substances in some areas, making it difficult for petroleum hydrocarbons to be efficiently degraded and resulting in the failure of ENA. Therefore, when using yeast extract for ENA, its migration and transformation in groundwater should be fully assessed to clarify its effective radius, and the environmental impact after use should be thoroughly evaluated. In view of this, for easily degradable light petroleum hydrocarbons (such as gasoline), pure inorganic nutrients may suffice to achieve ENA goals. In such scenarios, economic and easily migrating inorganic nutrients should be selected as ENA materials whenever possible. Meanwhile, the monitoring of organic nitrogen concentrations should be strengthened, especially when organic nitrogen is used as a nitrogen source. It is necessary to evaluate the transformation relationship between organic nitrogen and inorganic nitrogen, so as to clarify the nitrogen transformation mechanisms during the ENA process and determine the precise dosage of ENA materials. Apart from stimulating biological ENA, solubilization is also an ENA method that may promote contaminant degradation by increasing the microorganism’s exposure to contaminants [83]. To this end, earlier in this study, an attempt was made to conduct solubilization–microbial stimulation ENA experiments using high-concentration gasoline (with an NAPL phase). However, there was no significant difference between the treatment groups and the control group. This may be because petroleum hydrocarbons with a smaller number of carbon atoms and higher solubility (such as gasoline and benzene series compounds) have greater biotoxicity [84]. Excessively high concentrations of petroleum hydrocarbons are not conducive to biodegradation, and the application range of solubilized biodegradation may be limited to extremely insoluble contaminants.

It should be noted that this study simulated a facultatively anaerobic environment with a moderate amount of oxygen and moderate contaminant concentrations, which are widely distributed. The preferred solutions presented may not be applicable to other environments. Those studying sites with similar environmental conditions can refer to these preferred results, while those studying sites with significantly different environmental conditions can refer to this study to conduct simulation experiments and other work to select optimal ENA materials.

## 4. Conclusions

Drawing upon the current understanding of enhanced natural attenuation (ENA) technologies and the fundamental principles underlying groundwater contamination by petroleum hydrocarbons, this study aimed to identify the most effective ENA materials for addressing widely dispersed contamination plumes. The results showed that yeast extract containing nitrate, complex nitrogen fertilizers, and nitrate-containing formulated solutions were all effective ENA materials, which can not only stimulate the growth of microorganisms that degrade petroleum hydrocarbons but also enhance the mineralization of nutrient elements such as NPS, and even facilitate carbon sequestration. Among them, yeast extract demonstrated the best potential, both in terms of ENA effectiveness and microbial functional characteristics. However, how to effectively apply these materials in different field situations remains a technical challenge that needs to be addressed. In summary, this study provides preferred materials for the ENA of petroleum hydrocarbon contamination plumes in contaminated sites, offers theoretical and technical support for formulating site management and control measures, and contributes to the construction of clean cities.

## Figures and Tables

**Table 1:** The treatment groups of the ENA experiment.

Group	Material Categories	Treatment Performed	Manufacturer	Dosage to Be Added Each Time
NA	Natural attenuation	None	--	--
OC	Oxygen supply	Calcium peroxide	Nong xin ke ji	1600 mg/kg a
NY	Nitrate supply	Yeast extract	Solarbio^®^ (Cat#Y8020)	100 mg/kg [32]
NF		Nitrogenous fertilizer	Dugao^®^ Water-soluble fertilizer containing macronutrients (#25-10-20)	464 mg/kg b
NN		Self-compounding reagent containing Nitrate	In the study (see Table S1 for the specific formula)	20 mL/kg c

a. Maximum addition amount for a system with pH values not exceeding 8.5. b. The amount of nitrate added was calculated based on the stoichiometric relationship for nitrate-reducing hydrocarbon degradation. Due to the unknown composition, all nitrogen present was considered nitrate nitrogen. c. The amount of nitrate added was calculated based on the stoichiometric relationship for nitrate-reducing hydrocarbon degradation, while the addition of other elements is determined according to the literature [33].

**Table 2:** Comparison of actual degradation (AD) amount of petroleum hydrocarbons and theoretical degradation (TD) amount of nitrate reduction (unit: mg/kg).

Time (d)	6		12		18	
	TD	AD	TD	AD	TD	AD
NA	3.27 ± 0.17	77.95 ± 6.55	3.21 ± 0.06	83.76 ± 4.77	2.79 ± 0.38	136.71 ± 45.1
OC	−4.42 ± 1.94	−6.37 ± 29.33	0.18 ± 0.14	177.12 ± 67.8	0.02 ± 0	216.92 ± 16.27
NY	6.55 ± 0.04	138.61 ± 13.64	9.12 ± 0.33	587.16 ± 21.13	12.75 ± 0.04	613.43 ± 3.56
NF	0.01 ± 0.98	163 ± 40.14	7.86 ± 0.71	514.03 ± 30.5	12.32 ± 1.19	560.58 ± 0.41
NN	6.16 ± 2.77	132.61 ± 42.92	26.14 ± 9.1	474.27 ± 20.12	32.29 ± 17.95	562.53 ± 17.92

## Data Availability

The data that support the findings of this study are available from the corresponding author upon reasonable request.

## Supplementary Materials

The following supporting information can be downloaded at: https://www.mdpi.com/article/10.3390/microorganisms13071575/s1, Figure S1. Bar plot of the microbial community structure at the specie level; Figure S2: Abundance plot at the genus level for microorganisms containing the gene encoding formate C-acetyltransferase (EC 2.3.1.54); Table S1: The formula for self-compounding reagent; Table S2: The mass of extracted DNA from soil samples; Table S3: Supporting materials.

## References

[1] Sun L., Wang S.-w., Guo C.-j., Shi C., Su W.-C. (2022). Using pore-solid fractal dimension to estimate residual lnapls saturation in sandy aquifers: A column experiment. J. Groundw. Sci. Eng..

[2] Zhang H.Y., Han X., Wang G.C., Mao H.R., Chen X.L., Zhou L., Huang D.D., Zhang F., Yan X. (2023). Spatial distribution and driving factors of groundwater chemistry and pollution in an oil production region in the northwest China. Sci. Total Environ..

[3] Wijaya J., Park J., Yang Y.Y., Siddiqui S.I., Oh S. (2024). A metagenome-derived artificial intelligence modeling framework advances the predictive diagnosis and interpretation of petroleum-polluted groundwater. J. Hazard. Mater..

[4] Zhang M., Yang S., Zhang Z.F., Guo C.J., Xie Y., Wang X.Z., Sun L., Ning Z. (2023). Development and application of an integrated site remediation technology mix method based on site contaminant distribution characteristics. Appl. Sci..

[5] Dong X.Q., Yu K., Jia X.S., Zhang Y.Q., Peng X.X. (2022). Perchlorate reduction kinetics and genome-resolved metagenomics identify metabolic interactions in acclimated saline lake perchlorate-reducing consortia. Water Res..

[6] Giannetta M.G., Soler J.M., Queralt I., Cama J. (2023). Natural attenuation of heavy metals via secondary hydrozincite precipitation in an abandoned pb-zn mine. J. Geochem. Explor..

[7] Mulligan C.N., Yong R.N. (2004). Natural attenuation of contaminated soils. Environ. Int..

[8] Lu C.S., Xiu W., Yang B., Zhang H.Y., Lian G.X., Zhang T.J., Bi E.R., Guo H.M. (2024). Natural attenuation of groundwater uranium in post-neutral-mining sites evidenced from multiple isotopes and dissolved organic matter. Environ. Sci. Technol..

[9] Zhang S., Zhang C.-y., He Z., Chen L., Zhang F.-w., Yin M.-Y., Ning Z., Zhen S.-J. (2016). Application research of enhanced in-situ micro-ecological remediation of petroleum contaminated soil. J. Groundw. Sci. Eng..

[10] Sun Y., Yue G.S., Ma J. (2023). Transport and natural attenuation of benzene vapor from a point source in the vadose zone. Chemosphere.

[11] Ottosen C.F., Bjerg P.L., Kümmel S., Richnow H.H., Middeldorp P., Draborg H., Lemaire G.G., Broholm M.M. (2024). Natural attenuation of sulfonamides and metabolites in contaminated groundwater—Review, advantages and challenges of current documentation techniques. Water Res..

[12] Feng F., Yang Y., Liu Q.Y., Wu S.X., Yun Z.C., Xu X.J., Jiang Y.H. (2024). Insights into the characteristics of changes in dissolved organic matter fluorescence components on the natural attenuation process of toluene. J. Hazard. Mater..

[13] Juhasz A.L., Smith E., Waller N., Stewart R., Weber J. (2010). Bioavailability of residual polycyclic aromatic hydrocarbons following enhanced natural attenuation of creosote-contaminated soil. Environ. Pollut..

[14] Xu F., Bao J.Q., Liu Q., He X.X., Zhou Y.Q., Wang H., Xing J.M., Zhou L., Yuan J.F. (2024). Simultaneous natural attenuation of cr(vi) and nitrate in the hyporheic zone sediments from an upstream tributary of the Jinsha River in the Sichuan Basin. Sci. Total Environ..

[15] Sun Z.Y., Chu L.G., Wang X.H., Fang G.D., Liu C., Chen H., Gu C., Gao J. (2023). Roles of natural phenolic compounds in polycyclic aromatic hydrocarbons abiotic attenuation at soil-air interfaces through oxidative coupling reactions. Environ. Sci. Technol..

[16] Satoh H., Okabe S., Yamaguchi Y., Watanabe Y. (2003). Evaluation of the impact of bioaugmentation and biostimulation by in situ hybridization and microelectrode. Water Res..

[17] Zhou N., Yang Z.Y., Zhang J., Zhang Z.T., Wang H. (2024). The negative effects of the excessive nitrite accumulation raised by anaerobic bioaugmentation on bioremediation of pah-contaminated soil. Bioresour. Technol..

[18] Zhang M., Guo C., Shi C., Ning Z., Chen Z. (2021). A quantitative redox zonation model for developing natural attenuation-based remediation strategy in hydrocarbon-contaminated aquifers. J. Clean. Prod..

[19] Srivastava A., Valsala R., Jagadevan S. (2023). Biogeochemical modelling to assess benzene removal by biostimulation in aquifers containing natural reductants. Environ. Sci. Pollut. Res..

[20] Komlos J., Kukkadapu R.K., Zachara J.M., Jaffé P.R. (2007). Biostimulation of iron reduction and subsequent oxidation of sediment containing Fe-silicates and Fe-oxides: Effect of redox cycling on fe(iii) bioreduction. Water Res..

[21] Lian G.X., An Y.F., Sun J., Yang B., Shen Z.Y. (2024). Effects and driving mechanisms of bioremediation on groundwater after the neutral in situ leaching of uranium. Sci. Total Environ..

[22] Chen X.Y., Achal V. (2019). Biostimulation of carbonate precipitation process in soil for copper immobilization. J. Hazard. Mater..

[23] Chang S.H., Lin C.W., Cheng Y.S., Liu S.H. (2023). Effects of biodegradation, biotoxicity and microbial community on biostimulation of sulfolane. Chemosphere.

[24] Romantschuk M., Lahti-Leikas K., Kontro M., Galitskaya P., Talvenmäki H., Simpanen S., Allen J.A., Sinkkonen A. (2023). Bioremediation of contaminated soil and groundwater by in situ biostimulation. Front. Microbiol..

[25] Giovanella P., Duarte L.D., Kita D.M., De Oliveira V.M., Sette L.D. (2021). Effect of biostimulation and bioaugmentation on hydrocarbon degradation and detoxification of diesel-contaminated soil: A microcosm study. J. Microbiol..

[26] Liu H.A., Wang R.M., Luo M.Y., Xu C.H., Yu D.D., Zhan M.J., Long T., Yu R. (2024). Coupling of biostimulation and bioaugmentation for benzene, toluene, and trichloroethylene removal from co-contaminated soil. Water Air Soil Pollut..

[27] Ammeri R.W., Simeone G.D., Hidri Y., Abassi M.S., Mehri I., Costa S., Hassen A., Rao M.A. (2022). Combined bioaugmentation and biostimulation techniques in bioremediation of pentachlorophenol contaminated forest soil. Chemosphere.

[28] Mohammadi M., Bayat Z., Hassanshahian M., Mousavi M., Shekarchizadeh F. (2024). Microbial community response to biostimulation and bioaugmentation in crude oil-polluted sediments of the Persian Gulf. Environ. Res..

[29] Ning Z. (2019). Environmental Molecular Diagnosis of the Ammonium Affecting Petroleum Contaminants Degradation Mechanisms.

[30] Aldas-Vargas A., van der Vooren T., Rijnaarts H.H.M., Sutton N.B. (2021). Biostimulation is a valuable tool to assess pesticide biodegradation capacity of groundwater microorganisms. Chemosphere.

[31] Yuan F.Z., Zhao Y.Y., Dai Y.L., Yang W., Zhu J.Y. (2024). Effects of biostimulation-bioaugmentation on coastal microbial community in an in situ mesocosm system. J. Ocean Univ. China.

[32] Ning Z., Zhang M., Zhang N., Guo C., Hao C., Zhang S., Shi C., Sheng Y., Chen Z. (2022). Metagenomic characterization of a novel enrichment culture responsible for dehalogenation of 1, 2, 3-trichloropropane to allyl chloride. J. Environ. Chem. Eng..

[33] Zhang M., Ning Z., Guo C., Shi C., Zhang S., Sheng Y., Chen Z. (2023). Using compound specific isotope analysis to decipher the 1, 2, 3-trichloropropane-to-allyl chloride transformation by groundwater microbial communities. Environ. Pollut..

[34] Zwiener C., Frimmel F.H. (1998). Application of headspace gc/ms screening and general parameters for the analysis of polycyclic aromatic hydrocarbons in groundwater samples. Fresenius J. Anal. Chem..

[35] Lu R. (2000). Methods for Agrochemical Analysis of Soil.

[36] Vidyadharan P., Santhi C.K., Sethumadhavan A., Gopala S., Raghavan C., Urulangodi M. (2023). Functional assessment of DNA extraction methods from frozen human blood samples for sanger sequencing analysis. Cell. Mol. Biol..

[37] Nishii K., Möller M., Foster R.G., Forrest L.L., Kelso N., Barber S., Howard C., Hart M.L. (2023). A high quality, high molecular weight DNA extraction method for pacbio hifi genome sequencing of recalcitrant plants. Plant Methods.

[38] Ning Z., Sheng Y., Gan S., Guo C., Wang S., Cai P., Zhang M. (2024). Metagenomic and isotopic insights into carbon fixation by autotrophic microorganisms in a petroleum hydrocarbon impacted red clay aquifer. Environ. Pollut..

[39] Gan S., Zhang M., Zhou Y.H., Guo C.J., Yang S., Xie Y., Wang X.Z., Sun L., Ning Z. (2023). The effects of toluene mineralization under denitrification conditions on carbonate dissolution and precipitation in water: Mechanism and model. Appl. Sci..

[40] Ning Z., Guo C., Cai P., Zhang M., Chen Z., He Z. (2018). Geochemical evaluation of biodegradation capacity in a petroleum contaminated aquifer. China Environ. Sci..

[41] Ning Z., Cai P., Zhang M. (2024). Metagenomic analysis revealed highly diverse carbon fixation microorganisms in a petroleum-hydrocarbon-contaminated aquifer. Environ. Res..

[42] Gan S., Ning Z., Wang S., Sun W., Xu Z., Di H., Ti J., Guo C., Zhou Y., He Z. (2024). Identification of carbon fixation microorganisms and pathways in an aquifer contaminated with long-chain petroleum hydrocarbons. Water Environ. Res..

[43] Yang S., Zhang S.C., Ma S.C., Zhao S., Liu Z.W. (2024). Field demonstration of in situ slow-release oxygen chemicals coupled with microbial agents for injection to remediate btex contamination. Water.

[44] Zhou L., Qian Y., Chen J., Zhang Y., Zhou X. (2023). A critical review of solid peroxides in environmental remediation and water purification: From properties to field applications. Chem. Eng. J..

[45] Tomé D. (2021). Yeast extracts: Nutritional and flavoring food ingredients. ACS Food Sci. Technol..

[46] van Leeuwen J.A., Gerritse J., Hartog N., Ertl S., Parsons J.R., Hassanizadeh S.M. (2022). Anaerobic degradation of benzene and other aromatic hydrocarbons in a tar-derived plume: Nitrate versus iron reducing conditions. J. Contam. Hydrol..

[47] Song B.R., Tang J.C., Zhen M.N., Liu X.M. (2019). Effect of rhamnolipids on enhanced anaerobic degradation of petroleum hydrocarbons in nitrate and sulfate sediments. Sci. Total Environ..

[48] Jach M.E., Serefko A., Ziaja M., Kieliszek M. (2022). Yeast protein as an easily accessible food source. Metabolites.

[49] Shi K., Cheng H., Cornell C.R., Wu H., Gao S., Jiang J., Liu T., Wang A., Zhou J., Liang B. (2023). Micro-aeration assisted with electrogenic respiration enhanced the microbial catabolism and ammonification of aromatic amines in industrial wastewater. J. Hazard. Mater..

[50] Zhang Z., Zheng G.Y., Lo I.M.C. (2015). Enhancement of nitrate-induced bioremediation in marine sediments contaminated with petroleum hydrocarbons by using microemulsions. Environ. Sci. Pollut. Res..

[51] Li S.X., Liu F.J., Zheng F.Y., Huang X.G., Zuo Y.G. (2014). Risk assessment of nitrate and petroleum-derived hydrocarbon addition on *Contricriba weissflogii* biomass, lifetime, and nutritional value. J. Hazard. Mater..

[52] Di H., Zhang M., Ning Z., He Z., Liu C.L., Song J.J. (2024). A conceptual model for depicting the relationships between toluene degradation and fe(iii) reduction with different fe(iii) phases as terminal electron acceptors. Appl. Sci..

[53] Wu S.S., Hao W.Q., Yao Y., Li D.Q. (2023). Investigation into durability degradation and fracture of cable bolts through laboratorial tests and hydrogeochemical modelling in underground conditions. Tunn. Undergr. Space Technol..

[54] von Netzer F., Granitsiotis M.S., Szalay A.R., Lueders T. (2020). Next-generation sequencing of functional marker genes for anaerobic degraders of petroleum hydrocarbons in contaminated environments. Anaerobic Utilization of Hydrocarbons.

[55] Hammerle M., Hall E.A.H., Cade N., Hodgins D. (1996). Electrochemical enzyme sensor for formaldehyde operating in the gas phase. Biosens. Bioelectron..

[56] Ishizuka M., Ushio K., Toraya T., Fukui S. (1983). Formation of thieno 3,2-g pterines from the molybdenum cofactor. Biochem. Biophys. Res. Commun..

[57] Lambré C., Baviera J.M.B., Bolognesi C., Cocconcelli P.S., Crebelli R., Gott D.M., Grob K., Lampi E., Mengelers M., Mortensen A. (2024). Safety evaluation of the food enzyme 3-phytase from the non-genetically modified *Aspergillus niger* strain phy93-08. EFSA J..

[58] Pandey A., Szakacs G., Soccol C.R., Rodriguez-Leon J.A., Soccol V.T. (2001). Production, purification and properties of microbial phytases. Bioresour. Technol..

[59] Hove-Jensen B., Rosenkrantz T.J., Haldimann A., Wanner B.L. (2003). *Escherichia coli* phnn, encoding ribose 1,5-bisphosphokinase activity (phosphoribosyl diphosphate forming): Dual role in phosphonate degradation and nad biosynthesis pathways. J. Bacteriol..

[60] Hove-Jensen B., McSorley F.R., Zechel D.L. (2012). Catabolism and detoxification of 1-aminoalkylphosphonic acids: N-acetylation by the *PHNO* gene product. PLoS ONE.

[61] Agarwal V., Peck S.C., Chen J.H., Borisova S.A., Chekan J.R., van der Donk W.A., Nair S.K. (2014). Structure and function of phosphonoacetaldehyde dehydrogenase: The missing link in phosphonoacetate formation. Chem. Biol..

[62] Ruffolo F., Dinhof T., Murray L., Zangelmi E., Chin J.P., Pallitsch K., Peracchi A. (2023). The microbial degradation of natural and anthropogenic phosphonates. Molecules.

[63] Zhang M., Chen Q., Gong Z. (2024). Microbial remediation of petroleum-contaminated soil focused on the mechanism and microbial response: A review. Environ. Sci. Pollut. Res. Int..

[64] van der Ploeg J.R., Eichhorn E., Leisinger T. (2001). Sulfonate-sulfur metabolism and its regulation in *Escherichia coli*. Arch. Microbiol..

[65] Das S. (2023). Cell surface hydrophobicity and petroleum hydrocarbon degradation by biofilm-forming marine bacterium pseudomonas furukawaii pps-19 under different physicochemical stressors. J. Hazard. Mater..

[66] Lazzem A., Lekired A., Ouzari H.-I., Landoulsi A., Chatti A., El May A. (2024). Isolation and characterization of a newly chrysene-degrading *Achromobacter aegrifaciens*. Int. Microbiol..

[67] Alviz-Gazitua P., Durán R.E., Millacura F.A., Cárdenas F., Rojas L.A., Seeger M. (2022). Cupriavidus metallidurans ch34 possesses aromatic catabolic versatility and degrades benzene in the presence of mercury and cadmium. Microorganisms.

[68] Chayabutra C., Ju L.-K. (2000). Degradation of n-hexadecane and its metabolites by pseudomonas aeruginosa under microaerobic and anaerobic denitrifying conditions. Appl. Environ. Microbiol..

[69] Qu D., Zhao Y., Sun J., Ren H., Zhou R. (2015). Btex biodegradation and its nitrogen removal potential by a newly isolated pseudomonas thivervalensis mah1. Can. J. Microbiol..

[70] Liu X., Zhang Q., Yang X., Wu D., Li Y., Di H. (2023). Isolation and characteristics of two heterotrophic nitrifying and aerobic denitrifying bacteria, achromobacter sp. Strain hnds-1 and enterobacter sp. Strain hnds-6. Environ. Res..

[71] Zhang Z., Sun J., Guo H., Wang C., Fang T., Rogers M.J., He J., Wang H. (2021). Anaerobic biodegradation of phenanthrene by a newly isolated nitrate-dependent *Achromobacter denitrificans* strain phen1 and exploration of the biotransformation processes by metabolite and genome analyses. Environ. Microbiol..

[72] Strohm T.O., Griffin B., Zumft W.G., Schink B. (2007). Growth yields in bacterial denitrification and nitrate ammonification. Appl. Environ. Microbiol..

[73] Liu X., Li Z.W., Zhang C., Tan X.J., Yang X., Wan C.L., Lee D.J. (2020). Enhancement of anaerobic degradation of petroleum hydrocarbons by electron intermediate: Performance and mechanism. Bioresour. Technol..

[74] Caffrey S.M., Voordouw G. (2010). Effect of sulfide on growth physiology and gene expression of desulfovibrio vulgaris hildenborough. Antonie Leeuwenhoek.

[75] Korte H.L., Fels S.R., Christensen G.A., Price M.N., Kuehl J.V., Zane G.M., Deutschbauer A.M., Arkin A.P., Wall J.D. (2014). Genetic basis for nitrate resistance in *Desulfovibrio* strains. Front. Microbiol..

[76] Mahendran B., Choi N.-C., Choi J.-W., Kim D.-J. (2006). Effect of dissolved oxygen regime on growth dynamics of *Pseudomonas* spp. during benzene degradation. Appl. Microbiol. Biotechnol..

[77] Pinel-Cabello M., Wasmund K., Soder-Walz J.M., Vega M., Rosell M., Marco-Urrea E. (2024). Divergent dual c-h isotopic fractionation pattern during anaerobic biodegradation of toluene within *Aromatoleum* species under nitrate-reducing conditions. Environ. Pollut..

[78] Becker P., Wüensch D., Wöhlbrand L., Neumann-Schaal M., Schomburg D., Rabus R. (2023). The catabolic network of *Aromatoleum aromaticum* ebn1t. Microb. Physiol..

[79] Semenova E.M., Grouzdev D.S., Sokolova D.S., Tourova T.P., Poltaraus A.B., Potekhina N.V., Shishina P.N., Bolshakova M.A., Avtukh A.N., Ianutsevich E.A. (2022). Physiological and genomic characterization of *Actinotalea subterranea* sp. Nov. From oil-degrading methanogenic enrichment and reclassification of the family *Actinotaleaceae*. Microorganisms.

[80] Kuznetsov B.B., Ivanovsky R.N., Keppen O.I., Sukhacheva M.V., Bumazhkin B.K., Patutina E.O., Beletsky A.V., Mardanov A.V., Baslerov R.V., Panteleeva A.N. (2011). Draft genome sequence of the anoxygenic filamentous phototrophic bacterium *Oscillochloris trichoides* subsp. Dg-6. J. Bacteriol..

[81] Mechelke M., Koeck D.E., Broeker J., Roessler B., Krabichler F., Schwarz W.H., Zverlov V.V., Liebl W. (2017). Characterization of the arabinoxylan-degrading machinery of the thermophilic bacterium *Herbinix hemicellulosilytica*-six new xylanases, three arabinofuranosidases and one xylosidase. J. Biotechnol..

[82] Wei Y., Chen Y., Cao X., Xiang M., Huang Y., Li H. (2024). A critical review of groundwater table fluctuation: Formation, effects on multifields, and contaminant behaviors in a soil and aquifer system. Environ. Sci. Technol..

[83] Rahmani A.M., Gahlot P., Moustakas K., Kazmi A., Ojha C.S.P., Tyagi V.K. (2022). Pretreatment methods to enhance solubilization and anaerobic biodegradability of lignocellulosic biomass (wheat straw): Progress and challenges. Fuel.

[84] Heipieper H.J., Martínez P. (2018). Toxicity of hydrocarbons to microorganisms. Handbook of Hydrocarbon and Lipid Microbiology.

