# Supplementary material for: Enhanced Natural Attenuation of Gasoline Contaminants in Groundwater: Applications and Challenges of Nitrate-Stimulating Substances
Source: Microorganisms. 2025 Jul 4;13(7):1575. doi: 10.3390/microorganisms13071575 (PMC12298856; doi:10.3390/microorganisms13071575)
Supplement: Supplementary file 1 [file microorganisms-13-01575-s001.zip › Supporting materials-R2-final version.docx]

Supporting materials

Table S1. The formula for self-compounding reagent

| Formula | | The trace element solution | |
| --- | --- | --- | --- |
| Chemical component | Concentration (g/L) | Chemical component | Concentration (g/L) |
| KNO_3_ | 45.06 | FeSO_4_⋅4H_2_O | 1.8 |
| NH_4_Cl | 2.39 | CoCl_2_⋅6H_2_O | 0.25 |
| KH_2_PO_4_ | 0.61 | CuCl_2_⋅2H_2_O | 0.01 |
| Na_2_SO_4_ | 0.63 | NiCl⋅6H_2_O | 0.01 |
| Trace element solution | 10 | MnCl_2_⋅4H_2_O | 0.7 |
|  |  | ZnCl_2_ | 0.1 |
|  |  | H_3_BO_3_ | 0.5 |
|  |  | (NH_4_)_6_Mo_7_O_24_⋅4H_2_O | 0.1 |

Table S2. The mass of extracted DNA from soil samples

| Sample | The mass of soil sample used for DNA extraction (g) | The mass of extracted DNA (μg) |
| --- | --- | --- |
| Initial-0d | 1.5 | 0.06 |
| NA-6d | 1.31 | 0.06 |
| NA-12d | 1.45 | 0.06 |
| NA-18d | 2.16 | 0.01* |
| OC-6d | 2.55 | 0.03 |
| OC-12d | 0.63 | 0.06 |
| OC-18d | 2.07 | 0* |
| NY-6d | 1.32 | 0.09 |
| NY-12d | 1.29 | 0.14 |
| NY-18d | 0.55 | 0.1 |
| NF-6d | 0.59 | 0.09 |
| NF-12d | 0.6 | 0.07 |
| NF-18d | 1.4 | 0.07 |
| NN-6d | 0.64 | 0.06 |
| NN-12d | 0.56 | 0.06 |
| NN-18d | 1.61 | 0.08 |

* The total amount of DNA is too low to proceed with metagenomic sequencing


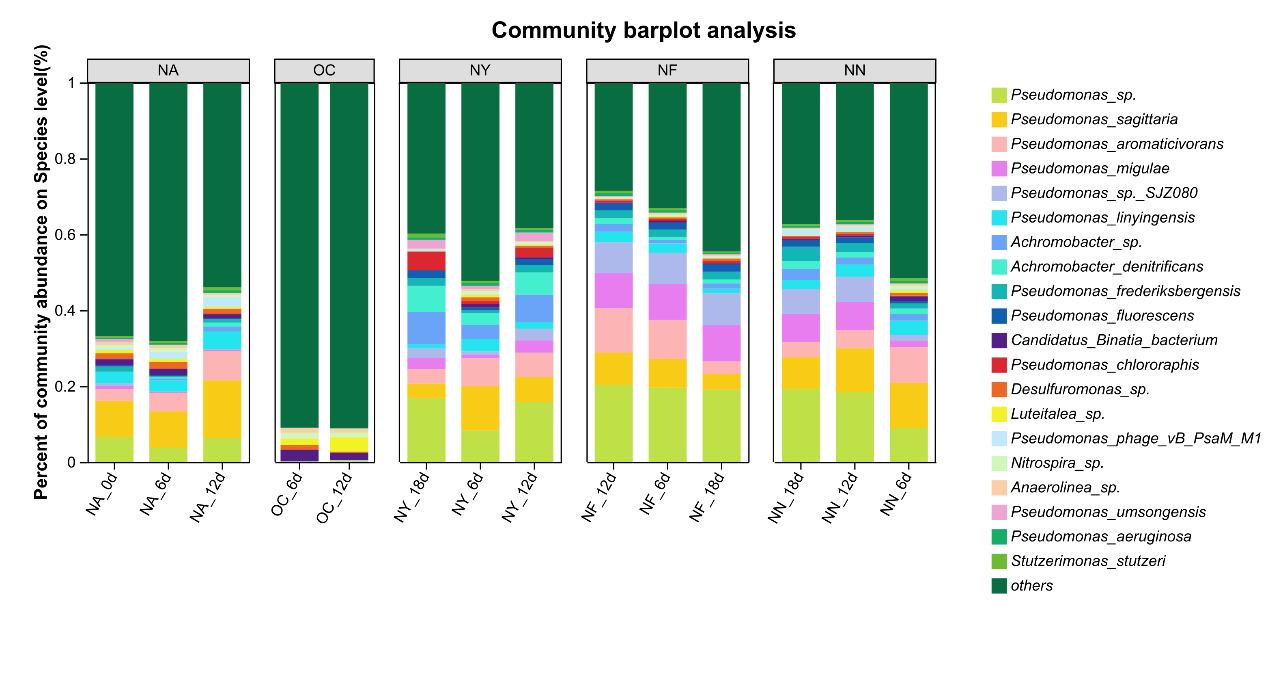


Figure S1. Bar plot of the microbial community structure at the specie level


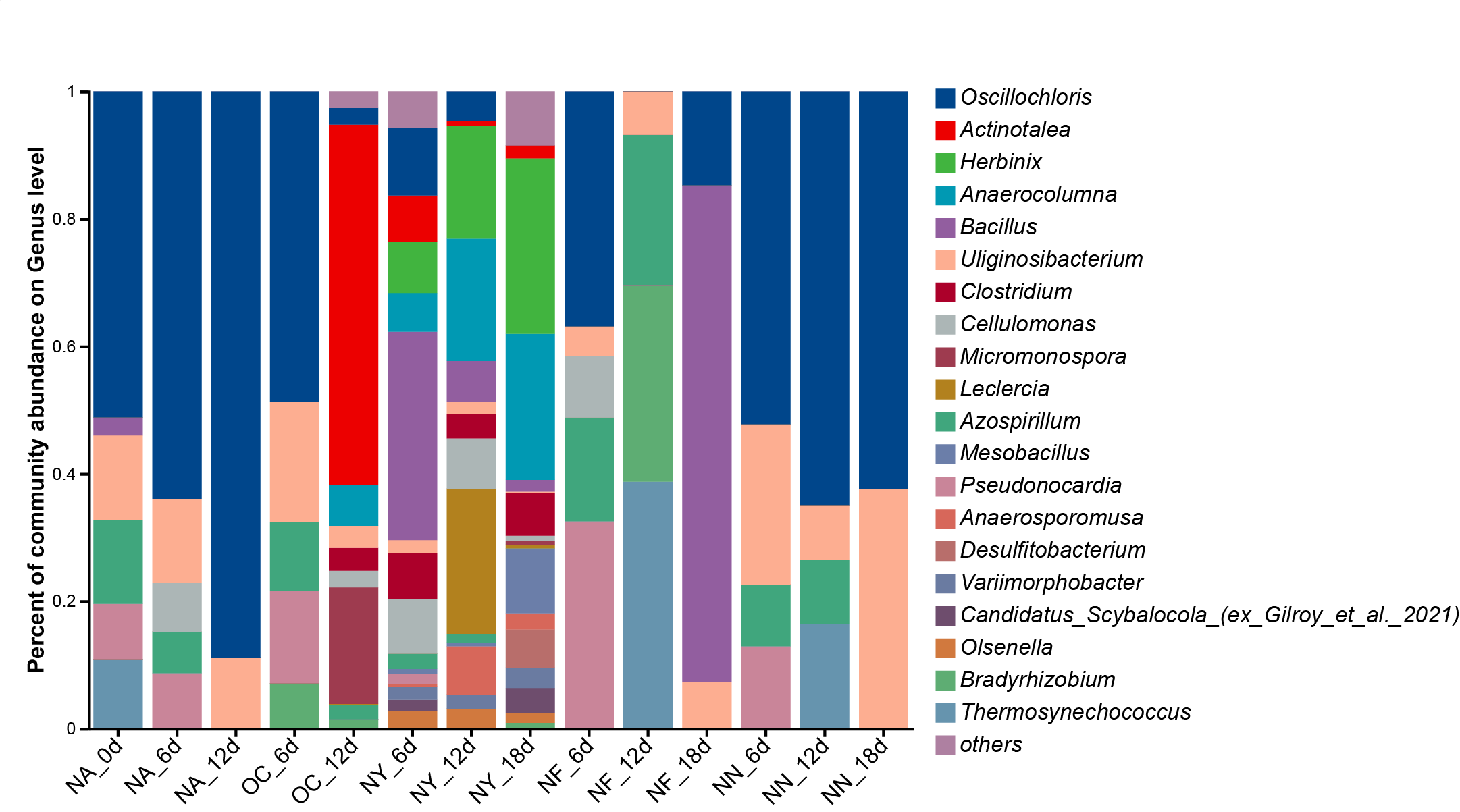


Figure S2. Abundance plot at the genus level for microorganisms containing the gene encoding formate C-acetyltransferase (EC 2.3.1.54)
